# A nontuberculous mycobacterium could solve the mystery of the lady from the Franciscan church in Basel, Switzerland

**DOI:** 10.1186/s12915-022-01509-7

**Published:** 2023-02-07

**Authors:** Mohamed S. Sarhan, Christina Wurst, Alexandar Tzankov, Andreas J. Bircher, Holger Wittig, Thomas Briellmann, Marc Augsburger, Gerhard Hotz, Albert Zink, Frank Maixner

**Affiliations:** 1 Eurac Research – Institute for Mummy Studies, 39100 Bolzano, Italy; 2 Institute of Medical Genetics and Pathology, University Hospital Basel, University of Basel, 4031 Basel, Switzerland; 3 Department of Allergology, University Hospital Basel, 4031 Basel, Switzerland; 4 Faculty of Biomedical Sciences, Università della Svizzera italiana, Lugano, Switzerland; 5 Department of Biomedical Engineering, Institute of Forensic Medicine, University of Basel, 4056 Basel, Switzerland; 6 Citizen Science Basel; formerly Institute of Forensic Medicine, Forensic Chemistry and Toxicology, University of Basel, 4056 Basel, Switzerland; 7 University Center of Legal Medicine, Lausanne, Geneva, Switzerland; 8 Natural History Museum Basel, 4051 Basel, Switzerland; 9 Integrative Prehistory and Archaeological Science, University of Basel, 4056 Basel, Switzerland

**Keywords:** Nontuberculous mycobacteria (NTM), Ancient DNA (aDNA), Bacteriophage, Syphilis, Brain infections, Mycobacteriosis, Franciscan church mummy, Anna Catharina Bischoff (ACB), De novo assembly

## Abstract

**Background:**

In 1975, the mummified body of a female has been found in the Franciscan church in Basel, Switzerland. Molecular and genealogic analyses unveiled her identity as Anna Catharina Bischoff (ACB), a member of the upper class of post-reformed Basel, who died at the age of 68 years, in 1787. The reason behind her death is still a mystery, especially that toxicological analyses revealed high levels of mercury, a common treatment against infections at that time, in different body organs. The computed tomography (CT) and histological analysis showed bone lesions in the femurs, the rib cage, and the skull, which refers to a potential syphilis case.

**Results:**

Although we could not detect any molecular signs of the syphilis-causing pathogen *Treponema pallidum* subsp*. pallidum*, we realized high prevalence of a nontuberculous mycobacterium (NTM) species in brain tissue sample. The genome analysis of this NTM displayed richness of virulence genes and toxins, and similarity to other infectious NTM, known to infect immunocompromised patients. In addition, it displayed potential resistance to mercury compounds, which might indicate a selective advantage against the applied treatment. This suggests that ACB might have suffered from an atypical mycobacteriosis during her life, which could explain the mummy’s bone lesion and high mercury concentrations.

**Conclusions:**

The study of this mummy exemplifies the importance of employing differential diagnostic approaches in paleopathological analysis, by combining classical anthropological, radiological, histological, and toxicological observations with molecular analysis. It represents a proof-of-concept for the discovery of not-yet-described ancient pathogens in well-preserved specimens, using de novo metagenomic assembly.

**Supplementary Information:**

The online version contains supplementary material available at 10.1186/s12915-022-01509-7.

## Background

**Fig. 1:**
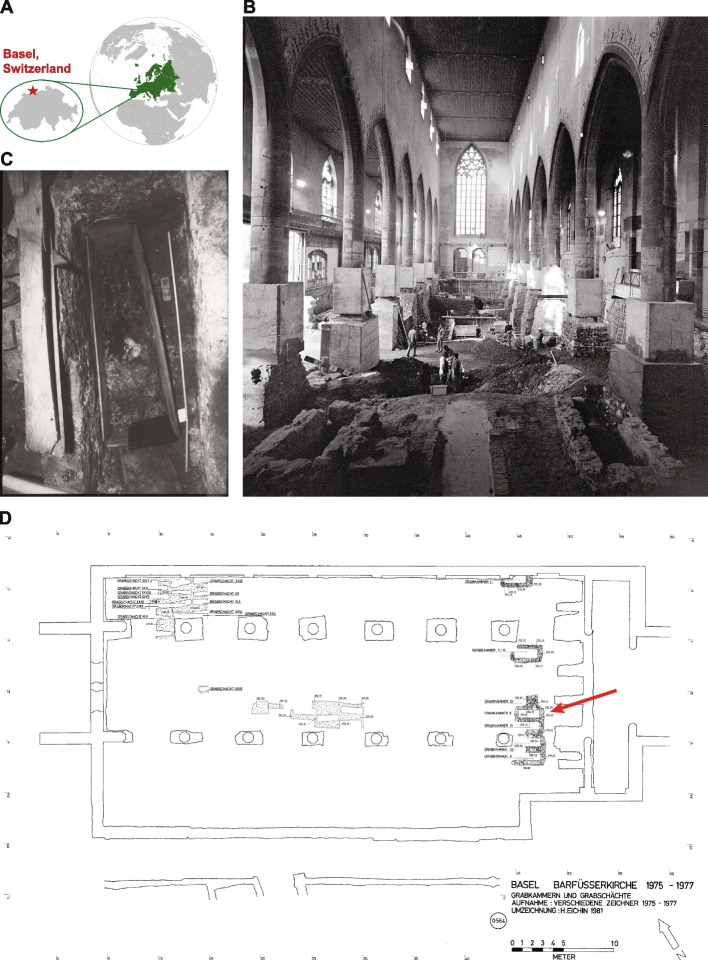
Description of the mummy’s finding site. **A** Map of Europe with zoom-in on Basel in Switzerland. **B** The Franciscan church during the renovation in 1975. **C** Photograph showing the first glance on the mummy tomb; recognizable are the overlapped hands. **D** The location of the burial chamber (indicated by red arrow) inside the Franciscan church (© Archäologische Bodenforschung, 1975/6 – Plan “Grabkammer und Grabschächte (G564)”, modified by H. Eichin 1981)

In 1975, a mummified corpse of a female individual was found in the Franciscan church (also known as the Barfüsser church) in Basel, Switzerland, during an excavation by the Archäologischen Bodenforschung Basel-Stadt in the church (Fig. 1A, B). The mummy’s coffin was encountered in a brick grave at a prominent position in the church (Fig. 1C, D), in front of the choir, along with another coffin that contained another human skeleton [1]. Genealogic studies and molecular analyses unveiled the mummy’s identity identifying her as Anna Catharina Bischoff (ACB, 29.03.1719-30.08.1787), a member of the upper class of post-reformed Basel, who died at the age of 68 years [2, 3]. By checking historical records of the church, it turned out that the mummy had been discovered earlier during the nineteenth century; then, due to ethical concerns, it has been reburied where it was again found in 1975 [2]. During this reburial, the coffin had been covered with soil (Fig. 1C).

The diseases and probable causes of death of ACB are still unknown, especially since the toxicological analyses of the mummy revealed high levels of mercury in different body organs [4], which is assumed to be the reason behind the mummification of the corpse (the antibacterial effect of mercury can slow down putrefaction), in addition to the dry conditions in the masonry grave shaft and the high salt concentration of the surrounding soils. Since mercury inhalation was a common treatment against infections, particularly syphilis [5], it was believed that she might have suffered from syphilis during her lifetime. This assumption was further supported by the computed tomography (CT) and histological results, which showed suggestive bone lesions in the skull, but degenerative lesions in femurs and rib cage. However, these lesions were histologically considered equivocal respecting late sequelae of syphilis infection, which led to additional differential diagnoses of a possible tuberculosis (TB) or Paget’s disease [6].

In this study, we aimed to investigate molecularly whether the mummy could have suffered from syphilis, by carrying out a comprehensive shotgun metagenomic analysis on different body organs, in order to detect possible DNA traces of the causing pathogen, *Treponema pallidum* subsp. *pallidum*, or other pathogens that might have led to the bone lesions or might be linked to the mercury treatment.

## Results

### Human mitochondrial DNA analysis of the samples confirms their origin

Initially, we analyzed the mitochondrial DNA of all tissues in order to confirm that they contain authentic DNA. All the samples taken from the mummy showed the same mitochondrial haplotype (i.e., U5a1+!16192) as previously reported [3], except for the skull bone sample, which showed additional background contamination with other human DNA (Additional file 1: Table S1) [3]. Interestingly, analysis of the human DNA of the maggots (Sample ID 3169), which were collected from underneath the mummy, revealed the same mitochondrial haplotype as of the mummy, indicating initial feasting on the decayed flesh of the mummy [7] or potential diffusion of mummy’s DNA into the surroundings [8]. The other control samples, coming from the skeletons of other individuals from the same tomb, showed two different mitochondrial haplotypes (Additional file 1: Table S1).

### Metagenomic analysis did not reveal any *Treponema* genomes but unusual high prevalence of *Mycobacteriaceae* in brain tissue

**Fig. 2:**
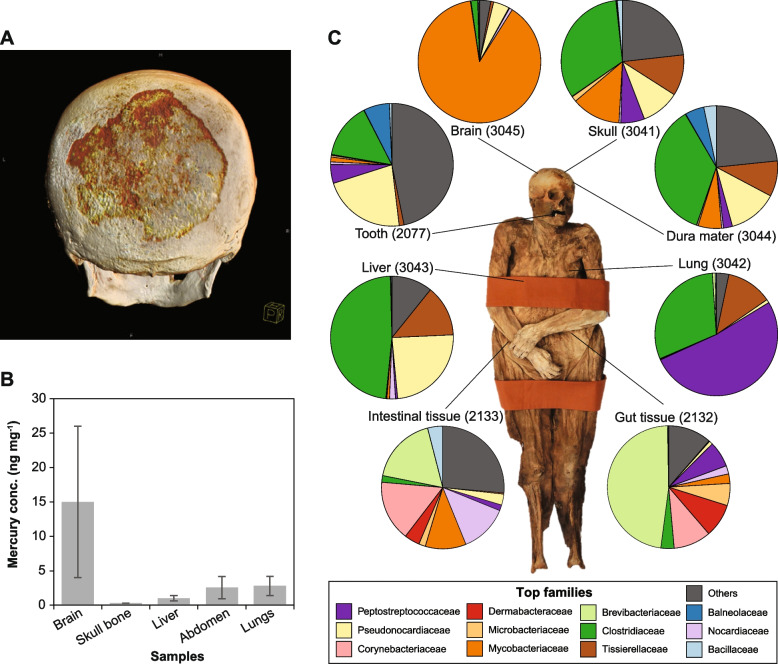
Overview on the radiological, toxicological, and microbiological characteristics of the ACB mummy. **A** Computed tomography (CT)-based three-dimensional reconstruction of the skull. Notice the darker colors which represent lower x-ray densities than healthy bone. Copyright: Holger Wittig, Institute of Forensic Medicine, University of Basel. **B** Concentration of elemental mercury in different body samples, where the error bars refer to the standard errors. **C** Relative abundances of the top 12 microbial families on different body tissues as inferred by number of shotgun-metagenomic reads compared against the nr-database (please refer to the “Methods” section for details). Numbers in parentheses refer to sample IDs (please refer to Additional file 1: Table S1 for further details)

Driven by the radiological and histological observations and the toxicological analysis (Fig. 2A, B), we carried out a shotgun metagenomic screening of different samples from different body parts of the mummy, representing tissues where the infection can be expected to occur (please refer to Additional file 1: Table S1 for details). Although most of the tissues displayed overwhelming prevalence of postmortem microbial communities, e.g., Clostridia, we were still able to spot some tissue-specific taxa, particularly in the gut (*Turicibacter sanguinis* and *Ruminococcus gnavus*) and the tooth (e.g., *Prevotella denticola* and *Actinomyces dentalis*) samples (Additional file 1: Table S2). Additionally, we did not find any metagenomic reads assigned to the syphilis-causing pathogen *Treponema pallidum* subsp*. pallidum* (Additional file 2: Figure S1), nor even the containing-family *Spirochaetaceae*, except for the tooth sample, which displayed presence of other *Treponema* species, e.g., *T. socranskii*, *T. denticola*, and *T. maltophilum*, which are all known to be linked to periodontitis and being members of the oral microflora [9, 10].

Unexpectedly, the brain tissue displayed exceptional high abundance of the family *Mycobacteriaceae*, representing more than 80% of the total microbial metagenomic reads (Fig. 2C, Additional file 2: Figure S2) [11–13]. In this respect, it is important to mention that the toxicological analysis displayed the highest mercury concentrations in the brain samples, i.e., up to 28 ng × mg^−1^ tissue material (Fig. 2B), since the brain is the target organ in the uptake or administration of elemental mercury. This opens a question on whether these two observations are correlated.

### De novo assembly revealed a nontuberculous mycobacterium (NTM) in the brain

**Fig. 3:**
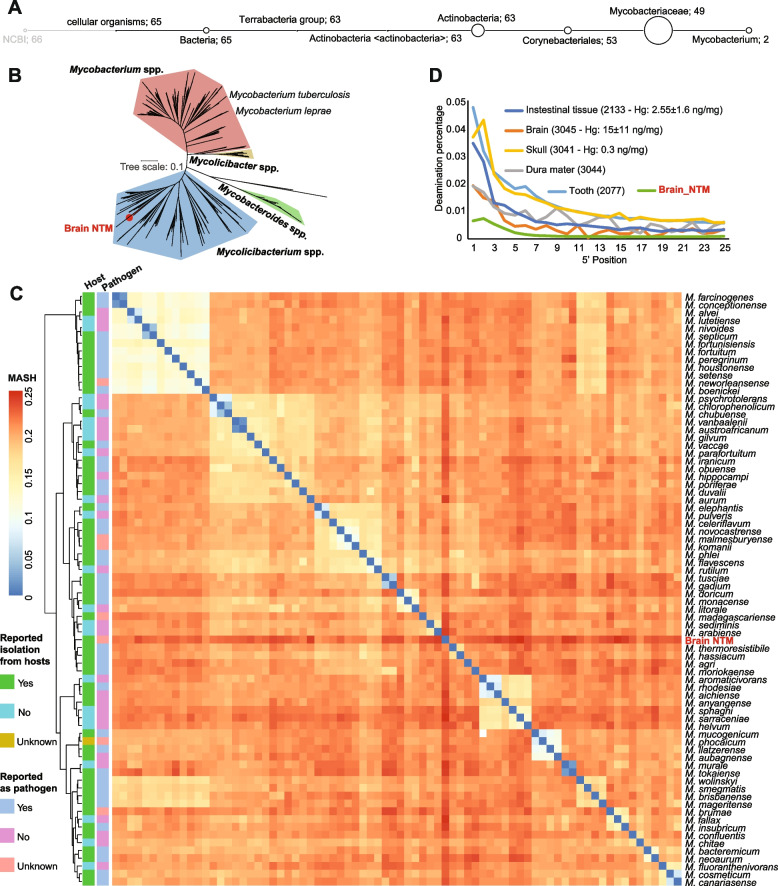
Genome-level taxonomic assignment of the brain bacterium. **A** Taxonomic assignment of the brain NTM contigs as assigned by searching against the NCBI-nt database, using BLASTn. The number of the assigned contigs is shown next the taxon names based on the lowest common ancestor (LCA) as determined by MEGAN (please refer to the “Methods” section for further details). **B** Unrooted phylogenetic tree of the family *Mycobacteriaceae*, including a single representative genome of each species, based on PhyloPhlAn marker genes. The background colors of the clades refer to: red, *Mycobacterium* spp.; yellow, *Mycolicibacter* spp.; green, *Mycobacteroides* spp.; and blue, *Mycolicibacterium* spp. **C** Heatmap-based on MASH distances of all characterized species’ genomes within the genus *Mycolicibacterium* including the genome of the brain bacterium (highlighted in bold red font). The heatmap annotations to the left of the heatmap refers to whether the microbe was isolated from a host or was reported as a human pathogen. For further details on the isolation sources, please refer to the Additional file 1: Table S6. **D** Damage plots of human DNA of different tissues as well as the brain NTM. The damage plots were generated considering the mapped reads of the indicated tissues different body tissues (i.e., tooth, intestinal tissues, skull, dura mater, and brain) against human genome (hg19) while the brain NTM was generated considering the brain metagenomic reads mapped against the brain NTM assembled genome. Ancient DNA damage represented by the terminal substitution of Cytosine to Thymine at the 5′ ends of the DNA fragments. The labels in parenthesis refer to sample ID and the mercury concentrations ± standard error. For further information on the read lengths distribution please refer to Additional file 2: Figure S3 [14, 15]. Note: The human DNA are showing variable levels of ancient DNA damages, despite of being of the same individual. The lowest levels of the human DNA damages are in the brain and dura mater samples, which goes with the abundance of the brain NTM and the mercury concentrations as well

Based on the high abundance of *Mycobacteriaceae* in the brain, we performed a de novo metagenomic assembly on the brain metagenomic reads (for further information on assembly, please refer to the “Methods” section). We were able to resolve a high-quality metagenome-assembled genome (MAG, 99.5 % completeness and < 0.5 % contamination, as estimated by CheckM), belonging to a nontuberculous mycobacterium (NTM) species (Fig. 3A, B, Additional file 1: Table S3). Interestingly, more than 57% of the brain metagenomic reads were mapped against the assembled genome (Additional file 1: Table S4). The genome constituted of 66 contigs of total size of ~ 4.8 Mb and mean coverage of 185.9 ± 45.4 × (Additional file 1: Table S5).

Considering that the genus *Mycobacterium* has undergone major phylogenomic-based taxonomic reassignments and rearrangements [16, 17], we used species-representative genomes of the whole *Mycobacteriaceae* family in order to gain an in-depth taxonomic characterization of our discovered genome within it, using PhyloPhlAn marker genes (please refer to the “Methods” section). The resulting phylogeny was highly congruent with the recently proposed taxonomy [16], having main clades representing the classical human pathogenic *Mycobacterium* spp., *Mycobacteroides* spp., *Mycolicibacter* spp., and finally *Mycolicibacterium* spp., where our genome falls within (Fig. 3B). Further, we compared our genome with all characterized species within the genus *Mycolicibacterium* to find the closest relative within the genus. For this purpose, we carried out a pairwise genomic comparison between all species including ours, using the Mash distance tool [18]. The mash distances of the brain NTM genome is relatively distant from all the characterized species within the genus (minimum distance = 20.03%) and clusters close to *M. thermoresistibile*, *M. hassiacum*, *M. agri*, and *M. moriokaense* (Fig. 3C). Although the four species are known to be, like other members of the genus, environmental bacteria, they have previously been reported to cause infections in humans (Fig. 3C, and for detailed examples, please refer to Additional file 1: Table S6) [19–94]. Moreover, most of *Mycolicibacterium* spp. have been previously isolated from hosts (Additional file 1: Table S6).

### Genome-wide analysis indicates potential virulence of the brain NTM

Before subjecting the brain genome to further functional analysis, we checked for the authenticity of this bacterium (henceforth referred to as “brain NTM”), i.e., being ancient on the one hand, and on the other being exclusively present in the brain, not being a contaminant from other tissues or even from the environment surrounding the burial site. Therefore, we tested the terminal deamination levels on the metagenomic brain reads mapped to the genome of the brain NTM (the “Methods” section). We noticed very low levels of terminal C-to-T and G-to-A substitutions, even less than the human DNA damage in this tissue (Fig. 3D, Additional file 2: Figure S3), although the fragment length distribution of the brain NTM was lower than of the human autosomal DNA (Fig. 3D, Additional file 2: Figure S3). When we further compared the human autosomal DNA from different organs, we realized variable levels of ancient DNA (aDNA) damages, correlating with the variable concentrations of mercury in different organs.

**Fig. 4:**
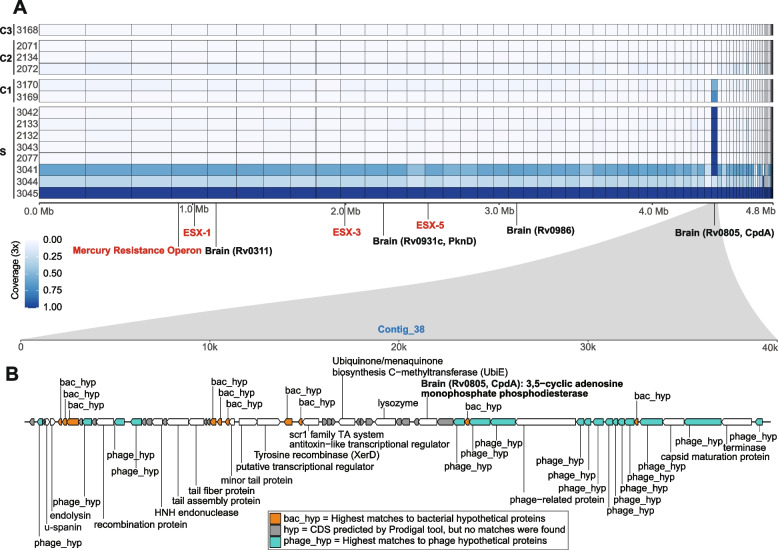
Genetic map of the brain bacterium genome. **A** The heatmap shows distribution of the DNA of the brain NTM in the mummy’s samples as well as other control samples; The numbers to the left refer to the sample ID as referred to in Additional file 1: Table S1, while the letters refer to the samples group as follows: S, the mummy’s tissue samples; C1, control samples that were in contact with the mummy; C2, samples that were taken from other individuals from the same burial site; C3, soil sample that was collected from inside the coffin of the mummy (please refer to Additional file 1: Table S1 and Additional file 2: Figure S4 for further details). The presence is shown by breadth values with minimum coverage of 3x per site. The contigs are arranged in a descending order from left to right (for details on the contigs, please refer to Additional file 1: Table S5). The loci referred to in bold red fonts are gene clusters/operons and are further detailed in Additional file 2: Figure S5, while those in black bold are genes that are known to be involved in crossing blood brain barrier (BBB) and brain invasion. **B** The genetic map of the contig 38, which is assumed to be a phage genome, as inferred by the tool PHASTER (for further details on the phage annotation, please refer to Additional file 1: Table S7)

To further assess the possibility of the brain NTM being an external contamination, we investigated in addition to the mummy tissues more samples representing the following (Additional file 2: Figure S4) [2]: (i) bone and tooth samples from other skeletons found in the same burial site (upper coffin); (ii) textile sample and maggots that were found on the mummy; and finally, (iii) soil samples that were found covering the upper parts of the mummy. After mapping all metagenomic reads of each of the aforementioned samples against the brain NTM genome and considering threshold of minimum 3x coverage (please refer to the “Methods” section for details), we did not find any sufficient breadth (i.e., > 50% of the genome covered at least 3 times) for any of the mummy’s samples, except for the skull bone and dura mater samples, which appeared to carry the bacterium, having average breadth values of > 70% (Fig. 4A). Thereby, we excluded the possibility of the external contamination and continued with the functional analyses.

We checked the overall virulence potential of the bacterium, by comparing all coding sequences (CDS) against the virulence factor database (VFDB) [95]. The genome contained three different clusters of type VII secretion systems (T7SSs), which are responsible for effector proteins in pathogenic and non-pathogenic mycobacteria, and help to survive in the host by evading the immune system [96]. Considering the genome of *M. tuberculosis* as a reference, our genome contained the core genes of the ESX-1 system as well as the full genes of the ESX-3 and ESX-4, with the same exact synteny arrangement (Additional file 2: Figure S5).

Since the NTM genome was exclusively present in the brain, we checked for the ability of the bacterium to invade the brain and cross the blood brain barrier (BBB). Be and colleagues identified the genes Rv0311, Rv0931, Rv0986, and Rv0805 (CpdA) in *M. tuberculosis* to be significantly involved in brain invasion and survival [97]. Indeed, we detected homologs of the four genes scattered throughout the genome (Fig. 4A). Moreover, it has been also previously reported that NTM can invade brain within circulating macrophages [98].

We additionally checked the brain NTM genome for the presence of toxin-antitoxin (TA) systems that are assumed to be contributing to the virulence of mycobacteria [99]. In comparison with other members of the genus *Mycolicibacterium*, and representatives of other known pathogenic mycobacteria, we realized that the brain NTM genome together with *M. tuberculosis* H37Rv were on the top in richness of TA systems (Additional file 2: Figure S6). Generally, TA systems are typically composed of a protein (toxin) and another antagonistic protein (antitoxin). Under stress conditions, the antitoxin, which is a labile molecule gets degraded, allowing the toxin molecule to halt essential cellular processes, leading to growth inhibition of bacteria [99]. Such process is reversible, which means that as soon as the conditions become favorable again, the antitoxin binds to the toxin and maintains essential cellular processes are restored. It is postulated that such TA systems play a great role in bacterial persistence under stress conditions, particularly in *M. tuberculosis* infections, which sometimes mediates dormant states tolerant to antibiotic treatment and the host’s immune response, i.e., latent tuberculosis. Analysis of transcriptomic profiles of antibiotic-induced persistence in *M. tuberculosis* revealed upregulation of 10 TA systems [100], which supports the assumptions of them being involved in pathogen persistence. In general, *M. tuberculosis* and ACB’s brain NTM contain remarkably higher numbers of TA systems, compared with other mycobacteria, so we can deduce that it may have caused a persistent infection, by entertaining a privilege over other microbes in terms of survivability and competitiveness in extreme conditions.

Finally, and in relation to the high mercury concentrations in the brain, we checked for the mercury resistance genes in the genome of the brain NTM in comparison with the mercury resistance operon of the *M. marinum* plasmid. We found in addition to the regulator protein gene and the core genes, i.e., mercuric reductase and alkylmercury lyase, other heavy metal resistance genes and antibiotic resistance genes (Additional file 2: Figure S5). The heavy metal resistance genes were mainly responsible for assimilation of copper, zinc, and arsenic, while the antibiotic resistance genes were directed against vancomycin, tetracycline, and beta-lactam antibiotics (Additional file 2: Figure S5).

### Evidence for a pre-infection transduction event that possibly increased the virulence

Although all mummy’s samples (except the brain, dura mater, and skull bones) seemed to be void of the brain NTM, they all displayed remarkable presence of one contig, i.e., contig 38, which indicates spread throughout the body, likely through the blood stream. To check the possibility that this contig might have been misplaced during the binning step, we went back to the metagenomic contigs and clustered them based on the coverage and the GC content, to whether this contig is an outlier to the rest of the genome. Considering both assembly methods, the contig was placed perfectly within the defined cluster of the brain NTM, showing similar coverage and GC content (Additional file 2: Figure S7). As further confirmation on the authenticity of the brain NTM, the contig 38 was completely absent from the samples of the other individuals and the soil sample. To explain the presence of this contig throughout the body, we hypothesized that it could be a circulating phage, since bacteriophages were shown to be present in the stool of patients with pulmonary diseases and to migrate through blood stream [101, 102]. Therefore, we screened for presence of viral sequences within our genome using the tool PHASTER (see the “Methods” section) and realized that the contig_38, which showed high prevalence in other mummy tissues, highly resembles phage genomes (Fig. 4B). Further, the CheckV tool displayed a high-quality viral genome with 91.35 completeness (Additional file 1: Table S8), having additionally other host/non-viral genes within the genome. By deeply annotating the contig, we found the basic structural proteins of phages, e.g., capsids, tail fibers, and portal proteins, in addition to other functional proteins, e.g., terminase, recombination proteins, and endolysin (Fig. 4B).

Interestingly, within this contig, there was the homologous gene of Rv0805 (CpdA), one of the genes that is involved in brain invasion and persistence. The presence of prophages has been recently reported in the genomes of clinical mycobacteria and assumed to aid in their virulence evolution. Moreover, when compared with environmental mycobacteriophages from PhagesDB, bacterial virulence genes were found to be enriched in the clinical mycobacterial prophages [103, 104]. This might indicate that the brain NTM has undergone a natural pre-infection transduction process that increased its virulence and enabled invasion and microbial survival in the brain.

## Discussion

The mummy of the Franciscan Church from Basel, Switzerland (ACB, 1719–1787) represents a unique example of multidisciplinary studied mummy [2]. Since the mummy is dated back to the eighteenth century, extensive historical, genealogical, and molecular investigations were necessary that resulted in reconstructing a family tree over 22 generations including nowadays living relatives [3]. The analyses showed that she belonged to a wealthy upper-class family, which might indicate that she might have had access to an advanced and special healthcare. The initial radiological and histological analyses of her mummified body indicated combination of different diseases, such as atherosclerosis and gallstones, and the toxicological analysis showed high concentrations of mercury in different body parts. Thus, studying the paleopathological status and the treatments she received provide insights into an important time in the European history, as she lived during the scientific revolution and before the onset of the industrial revolution.

One of the main intriguing findings was the high mercury concentration that has been revealed even at the mummy’s discovery time in 1975 [105] and was assumed to be one of the main reason for mummification. The presence of mercury also triggered some questions regarding her health status and in which context she had been exposed to these high concentrations of mercury. Historically, the beginning of mercury usage in the medical fields in Europe dates probably back to 1495, at the beginning of the first documented syphilis outbreak [106]. During that time, mercury had been already in use in the Arabic medicine to treat skin infections and leprosy [5, 107]. Therefore, it was adopted in Europe to treat syphilis and other infectious diseases by different methods of treatments, such as topical application, inhalation of mercury vapors, or even by ingestion of mercury salts [5, 108–110]. From the toxicological point of view, humans can get exposed to different forms of mercury, i.e., inorganic (e.g., mercury chloride), organic (e.g., methylmercury), or elemental (e.g., mercury vapors) [111]. The elemental form is absorbed by inhalation, passes from the lungs into the blood, crosses the blood-brain barrier and accumulates in the brain [112, 113]. While the inorganic forms, due to their lower liposolubility, were hypothesized to need to be accompanied with selenium (Se) that neutralizes the mercury’s toxicity to be able to access and cumulate in the brain [114]. On the other hand, mercury in its ionic forms is mainly accumulated in the kidney and liver. Considering that we found higher mercury concentrations in the brain than in the other body samples, we postulate that ACB was exposed to mercury vapors as a treatment for an extended time period. However, we cannot exclude that ACB was exposed to a treatment with an ointment containing colloidal elemental mercury or another inorganic mercury treatment, considering the correlation between the concentrations of the selenium and mercury, particularly in the brain of ACB (Additional file 2: Figure S8).

The classical explanation for the presence of mercury in the mummy would be the syphilis, which is mainly a sexually transmitted bacterial disease caused by *Treponema pallidum* subsp. *pallidum* [115]. Its symptoms are highly variable and can range from small chancres and nodular granulomatous skin lesion to late-onset prominent bone lesions [116]. Syphilis is also known as one of the “great imitators,” as it causes symptoms similar to other diseases making its diagnosis challenging [117]. For instance, in 1885, a bacillus bacterium was isolated from a syphilitic chancres [118, 119], which was assumed to be the causing pathogen, but later and by further research turned out to be the non-tuberculous *M. smegmatis* that causes very similar ulcers to those of *T. pallidum* subsp. *pallidum* [77]. In our case, we could not find any metagenomic reads that can be assigned to *T. pallidum*, and the only partially supporting clues would be the bone lesions unveiled by the radiological analysis with yet equivocal histopathology and the existence of mercury itself, where both are indirect evidence on syphilis. However, it might be useful in the future to consider employing a target enrichment capture approach particularly for those hardly to recover pathogens [120].

The other possible explanation for the mercury treatment can be deduced from the extraordinarily high abundance of *Mycobacteriaceae* in the brain of ACB as well as scattered incidence in the dura mater and the skull bone samples. By employing de novo metagenomic assembly, we resolved a near-full NTM genome. In general, NTM are considered as environmental bacteria, which inhabit different niches like soils and water. Many of the NTM were reported to cause human infections but remained neglected for decades until their recent remarkable emergence in clinical infections [121, 122]. NTM affect primarily human lungs, causing pulmonary disease, but can also infect other body parts, particularly in immunocompromised patients [123]. In the ancient metagenomics and paleopathology fields, NTM are usually mentioned within environmental contexts or as contaminants, due to the difficulty to confidently assign them as true pathogens like *M. tuberculosis* [124].

However, in this study, we opted to extensively analyze the genome of the brain NTM to explain their unusual existence and high abundance in the brain. The primary approach to check the DNA of a genome of whether it is ancient, is to analyze the ancient DNA damage pattern, i.e., terminal 5′C-to-T deamination. When we checked the damage pattern of the brain NTM, we found low damage levels (Fig. 3D). Interestingly, when we checked the damage of the human DNA of different organs of the mummy, we realized variability in the damage levels, negatively correlating with the mercury concentrations. For instance, the highest mercury concentrations were found in the brain samples, where we found the lowest human DNA damage (please refer to Fig. 3D for further examples). Despite of being a rare finding, this observation could explain the low damage levels on the brain NTM and highlights the inter-body variability of the DNA damages, which might open a discussion on using the ancient DNA damage as a primary tool for assessing modern contamination, particularly when dealing with unusually mummified materials.

In our case, the NTM contamination possibility can be toned down as soon as we consider the following: (i) the extraordinarily high abundance in the brain; (ii) the exclusive incidence in the brain and the close-by tissues and the absence from the other control samples as well as other organs of the mummy; (iii) the distribution of the putative phage within the mummy’s tissues and the absence from the control samples; (iv) the virulence potential of the genome particularly including genes that can enable crossing the BBB; (v) the richness of TA-systems that presumably enabled persistence under stress and unfavorable conditions; and, finally, (vi) the mercury-, heavy metal-, and antibiotic resistance genes of the bacterium. Therefore, we can gain confidence on the origin of the bacterium and its potential to be a brain pathogen that survived despite of the mercury treatment.

Overall, and based on the radiological, histological, toxicological, and molecular analyses of the ACB’s mummy, the current situation might suggest two different scenarios that explain her health status in relation to the high mercury concentrations. First, ACB suffered from syphilis and was exposed to a mercury treatment successfully, and *T. pallidum* was completely eradicated, while the NTM brain infection occurred later. Second, she suffered from an NTM, and probably showed symptoms similar to syphilis, and was therefore subjected to mercury treatment, but the pathogen survived the mercury due to its content of mercury and heavy metal resistance genes. The first scenario can be supported by the radiological findings, i.e., the bone lesions of the skull and the ribs; however, it is not supported at the molecular level. While the second scenario is highly supported by the molecular analysis, and radiological signs could also support this hypothesis.

## Conclusions

In conclusion, this study spots the light on one NTM as one of the, not only nowadays, cause of neglected diseases and infections, which might have been misdiagnosed as syphilis or tuberculosis during the eighteenth century and may still be overlooked or misinterpreted nowadays in paleopathological studies due to the guided interest in more common and better-known diseases. The study of this mummy exemplifies the importance of employing differential diagnostic approaches in paleopathological analysis by combining classical anthropological and radiological observations with molecular investigations. It also demonstrates the value of employing comparative metagenomic approaches in analyzing multiple samples from one individual. Finally, the study shows the value of employing de novo metagenomic assembly in recovering extinct and not-yet described ancient genomes, in well-preserved specimens, and gaining better insights on their phylogenetic and functional characteristics that are often beyond the potential of other tools.

## Methods

### Mummy sampling

The analyzed tissues and bones have been sampled at the Natural History Museum of Basel (Additional file 1: Table S1). The sampled tissues were checked visually and histologically to confirm their tissue of origin. Additional control samples were taken from the soil surrounding the mummy and the textile found underneath the body. Moreover, bone and teeth samples were taken from the other skeletons found in another coffin in the same burial site (please refer to Additional file 2: Figure S4).

### DNA extraction, library preparation, and DNA sequencing

Amounts of 9–200 mg of the ancient materials or the control samples were used for DNA extraction, following the protocol described by Maixner and colleagues [125]. DNA extracts were quantified using QUANTUS (Promega, USA); then, 20 μl of each extract were converted into double-indexed DNA libraries following a special protocol for highly degraded ancient DNA [126]. All previous steps were carried out in the ancient DNA laboratory of the Eurac Research Institute for mummy studies in Bolzano, Italy. The double-indexed libraries were subjected to next generation DNA sequencing using HiSeqX (2 × 150 paired-end), resulting in sequencing depths of 9–51 × 10^6^ paired-end reads per sample (Additional file 1: Table S1).

### Processing of ancient DNA sequences

We used the tool fastp [127] to trim the adapters and low-quality reads and to merge paired-end reads with at least 10 nucleotides overlap. Then, the quality filtered merged reads were de-duplicated and filtered for minimum sequence length of 25 nucleotides, using SeqKit [128].

### Human DNA analysis

To check the authenticity of the analyzed materials, we included, as a reference, in our analyses the shotgun metagenomic dataset generated from a tooth sample that was used to reveal the identity and the mitochondrial haplogroup of the mummy (https://www.ebi.ac.uk/ena/browser/view/PRJEB44723 ) [3]. For the analysis, we mapped the quality-filtered deduplicated merged reads against the human reference genome (build hg19) [129] and the human reference mtDNA genome (rCRS) [130] using Burrows-Wheeler Aligner (BWA) [131]. Then, we used SAMtools to filter for minimum mapping quality of 30, QualiMap to generate basic mapping statistics [132], and mapDamage 2.0 [14] to quantify the percentages of C-to-T and G-to-A substitution of the mapped ancient DNA reads. In the case of mitochondrial DNA, we additionally used option “--*rescale*” to rescale the quality scores of the damaged mis-incorporated sites and the tool Schmutzi to estimate the level of contamination based on deamination patterns [133]. For haplogroup assignment, we first converted the rescaled bam files into variant calling format (VCF) and then employed HaploGrep 2.0 [134].

Moreover, and to confirm the sex of the mummy samples, we used the mapped human DNA reads to compute the karyotype frequency of X and Y chromosomes, using a Maximum likelihood method [135].

### General microbial profiling

To have an overview on the microbial composition of the samples, we used the search tool BLASTX of DIAMOND v2.0.13 [136] to compare our metagenomic reads, with the default parameters, against the nr-protein database [137]. Then, we used MEGAN v6.21.16 [11] to process the DIAMOND outputs and to assign the metagenomic reads to their lowest common ancestor (LCA), with the parameters “*--minPercentIdentity 97*” and “*--minSupport 10*”. Since the BLASTX compares nucleotides against amino acid sequences, we confined our LCA filters to the family-level to minimize the false positives. The number and percentages of assigned reads are summarized in Additional file 1: Table S4.

### De novo assembly of metagenomic reads

We used the quality-filtered unmerged reads of the brain sample (Eurac ID 3045, 26411722 pair-end reads) to perform de novo sequence assembly, using the metagenomic assemblers MEGAHIT [138] and SPAdes with “--meta” option [139]. All contigs shorter than 1000 nt were excluded from the downstream analyses. The metagenomic binners MetaBAT2 [140], MaxBin2 [141], and CONCOCT [142] were used to resolve potential genomes, based on similarity of abundance and tetranucleotide frequency. Then, the DAS Tool was used to resolve consensus bins from each assembler, independently (Additional file 2: Figure S7).

Further, we checked the completeness and contamination of the resulting bins using CheckM [143] and kept only the high-quality genomes (completeness > 90% and contamination < 5%). CheckM checks for the presence of single-copy genes (SCG) specific for each lineage. If the program finds more than one copy of any of those SCG, it considers this as a potential contamination. To calculate the strain heterogeneity, the program calculates the relatedness between the multiple copies of those SCG, based on amino acid similarity. Then, the function “*classify_wf*” of GTDB-Tk v1.5.0 (April 23, 2021) has been used, with default parameters, to classify the resulting bins taxonomically [144, 145].

As a result, two of the bins displayed identical taxonomic classification (g__*Mycobacterium* sp.) and MASH distance of < 0.01, each is resulting from different assembler, i.e., MEGAHIT and SPAdes. Therefore, we used SeqMan tool of DNASTAR [146] to reassemble the contigs of both bins, by aligning all contigs vs all contigs, in order to improve the assembly quality, e.g., N50 value and number of contigs. Finally, we ended up with near-full genome with 99.55% completeness and 0.45% contamination (Additional file 1: Table S3).

To check the taxonomy of the resulting contigs, we searched all the contigs against the NCBI-nt database [137]. Then, the module “*blast2rma*” of MEGAN v6.21.16 [11] with the parameters “*--minPercentIdentity 80*” has been applied to assign the contigs to their lowest common ancestor (LCA).

### Phylogenetic analysis

To classify the resulting genome phylogenetically, we used the PhyloPhlAn 3.0 tool [147] to retrieve representative genomes of each species within the family Mycobacteriaceae. Then, we built the phylogeny based on the 400 universal PhyloPhlAn marker genes using the option “*--diversity low --accurate*”. The configuration file included the following tools with the default parameters set by PhyloPhlAn: DIAMOND v2.0.13 [136], MAFFT v7.427 [148], trimAl v1.4.1 [149], FastTree v2.1.11 [150], and RAxML v8.2.12 [151].

Based on the PhyloPhlAn phylogenetic assignment, we retrieved representative genomes of all characterized species belonging to the genus *Mycolicibacterium* and added our brain bacterium genome, then calculated MASH distances all vs. all, using the “*phylophlan_metagenomic*” module of PhyloPhlAn 3.0 [18].

### Checking the brain bacterium in the control samples

To check the presence of the brain bacterium in other tissues than the brain or in the control samples, we mapped the quality-filtered reads of each sample against the sequence of the brain bacterium, using Bowtie2 [152], with the option “*--end-to-end*.” Then, we sorted and indexed the resulted bam files using SAMtools [153], including a minimum mapping quality of 30. We used the script CMSeq (https://github.com/SegataLab/cmseq) to calculate the depth and breadth of the mapping. Positions were considered as true covered positions if they were covered at least 3 times. Afterwards, we calculated the breadth per contig and plotted the breadth as a heatmap (Fig. 4).

Finally, we used the tool DamageProfiler [15] to check for ancient DNA damage patterns, i.e., C-to-T and G-to-A substitutions, resulting from cytosine deamination.

### Bacterial genome annotation

In addition to the genomes of the *Mycolicibacterium* spp., we included the genome of *Mycobacterium tuberculosis* H37Rv from the Genbank databases (NCBI Reference Sequence: NC_018143.2) and *Mycobacteroides abscessus* (NCBI Reference Sequence: NZ_CP034180.1), in the genome annotation analyses. We used the Prokka pipeline to annotate the genomes [154], implemented Prodigal for gene prediction [155], and RNAmmer to find ribosomal RNA genes [156].

To search for the virulence genes, we compared all coding sequences against the virulence factor database (VFDB) [95], using mmseqs2 with its default parameters. To annotate the type VII secretion system gene clusters, we manually extracted the regions from *M. tuberculosis* H37Rv and our brain bacterium genome, based on Prokka annotation and compared them in a pairwise manner, using the BLASTp (all vs. all). Visualization of gene synteny was done using Clinker [157]. Following the same previous approach, we compared the mercury resistance operon in our brain bacterium genome to the well-characterized mercury resistance operon of *M. abscessus*.

To find the genes that are potentially involved in brain invasion [97], we manually retrieved the genes (*Rv0311*,*Rv0805*, *Rv0931c*, and *Rv0986*) from the Mycobrowser database (https://mycobrowser.epfl.ch). The tool OrthoFinder [158] was used to find the homologous sequences in our brain bacterium genome as well as the other *Mycolicibacterium* spp. genomes, including the *M. tuberculosis* and *M. abscessus*.

To check the presence of toxin/antitoxin (TA) systems in the analyzed genomes, we compared all coding sequences inferred by Prokka against the TA database (TADB 2.0), and confined the analysis to the experimentally validated type II TA loci [159]. We used mmseq2 for comparison using default parameters.

### Viral genome annotation

To search for viral sequences within the genome of the brain bacterium, we used the tool PHASTER (PHAge Search Tool Enhanced Release) [160], which predicts and annotates phage genes with comparison to curated phage and bacterial gene databases (https://phaster.ca). Additionally, we used the tool CheckV [161] to check the quality and completeness of the potential phage genomes. Then, we used different approaches to perform functional annotation of the contig which has been assigned as phages: (i) we used Prokka standard annotation as described previously; (ii) we used the PHASTER annotation tool; (iii) we used the tool MicrobeAnnoatator [162] implementing a DIAMOND search against different databases (i.e., KEGG Orthology, KO; Enzyme Commission, E.C.; Gene Ontology, GO; Pfam; and InterPro); and finally (iv) we used the MMseqs2 protein search tool [163] against the IMG/VR v3 database, which includes genomes of cultivated and uncultivated viruses [164].

### Mercury determination

The concentrations of mercury were measured in samples by inductively coupled plasma system coupled to mass spectrometry (ICP-MS; 7700 Series; Agilent, Palo Alto) at the University center of legal medicine (Geneva, Switzerland). Prior to analysis, samples were diluted with aqua regia to dissolve even poorly soluble mercury salts. The solution contained 10 ng/mL Rhodium (Rh) and 10 ng/mL Indium (In) as internal standards. In addition, each analytical batch of study samples was processed with laboratory controls, including method blanks and standard reference materials to continuously monitor method performance.

### Graphical plotting

The following R packages were used to plot the data: “*ggplot2*,” “*pheatmap*,” “*webr*,” and “*moonBook*” in R-studio (https://www.rstudio.com).

## Supplementary Information

**Additional file 1: Table S1.** Sequencing basic statistics and mitochondrial haplogroup assignments. **Table S2.** Taxonomic classification on the bacterial species-level as estimated by mapping the metagenomic reads against the NCBI-nr database using DIAMOND/BLASTx mode and lowest common ancestor (LCA) assignment using MEGAN. **Table S3.** Basic assembly statistics and CheckM results of different assembly approaches. **Table S4.** Comparison of total number of bacterial-assigned reads, as estimated using DIAMOND search against NCBI-nr database and Brain_NTM-assigned reads by mapping. **Table S5.** The lengths, coverage, and GC content of the contigs of the brain NTM after combining SPAdes and MEGAHIT assemblies. **Table S6.** Metadata on the characterized members of the genus *Mycolicibacterium*. **Table S7.** Combined annotation of the contig_38. **Table S8.** CheckV results of the contig_38.

**Additional file 2: Figure S1.** NCBI taxonomy-based cladogram of the metagenomic reads mapped to the reference genome of *Treponema pallidum* subsp. *pallidum* (NZ_CP010561.1). **Figure S2.** Top bacterial families in the brain tissue as inferred by different taxonomic classifiers. **Figure S3.** Read lengths distribution of human DNA of different tissues as well as the brain NTM. **Figure S4.** Description of the analyzed samples. **Figure S5.** Virulence genes of the brain NTM. **Figure S6.** Toxin/Antitoxin (TA) Systems in the analyzed mycobacterial genomes. **Figure S7.** Metagenomic binning of Anna Catharina Bischoff’s (ACB) brain sample. **Figure S8.** Correlation analysis between concentrations of mercury (Hg) and Selenium (Se) in different body parts.

## Data Availability

Raw sequencing data are publicly available on the European Nucleotide Archive (ENA, Project ID: PRJEB44723) [165].
