# Supplementary material for: A nontuberculous mycobacterium could solve the mystery of the lady from the Franciscan church in Basel, Switzerland
Source: BMC Biol. 2023 Feb 7;21:9. doi: 10.1186/s12915-022-01509-7 (PMC9903526; doi:10.1186/s12915-022-01509-7)
Supplement: Supplementary file 2 — Additional file 2: Figure S1. NCBI taxonomy-based cladogram of the metagenomic reads mapped to the reference genome of Treponema pallidum subsp. pallidum (NZ_CP010561.1). Figure S2. Top bacterial families in the brain tissue as inferred by different taxonomic classifiers. Figure S3. Read lengths distribution of human DNA of different tissues as well as the brain NTM. Figure S4. Description of the analyzed samples. Figure S5. Virulence genes of the brain NTM. Figure S6. Toxin/Antitoxin (TA) Systems in the analyzed mycobacterial genomes. Figure S7. Metagenomic binning of Anna Catharina Bischoff’s (ACB) brain sample. Figure S8. Correlation analysis between concentrations of mercury (Hg) and Selenium (Se) in different body parts. [file 12915_2022_1509_MOESM2_ESM.docx]

**Supplementary figures**


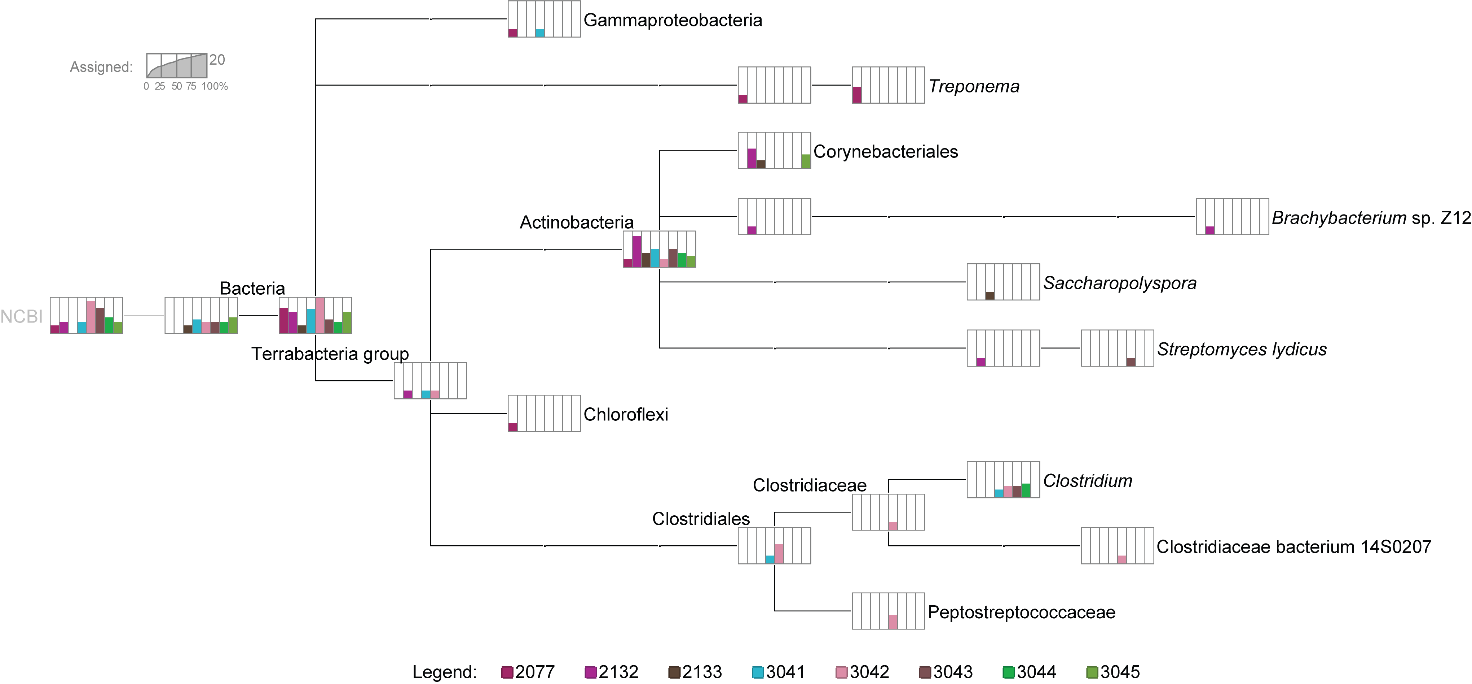


**Figure S1: NCBI taxonomy-based cladogram of the metagenomic reads mapped to the reference genome of *Treponema* *pallidum* subsp. *pallidum* (NZ_CP010561.1).** The shotgun metagenomic reads of all samples were initially mapped against the indexed reference genome using Burrows-Wheeler Aligner (BWA) with lenient parameters “bwa aln -n 0.01 -l 16”. After filtering the reads with a mapping quality < 30, the reads were exported into FASTA format. Reads were aligned against the NCBI-nt database, using the basic local alignment search tools (BLAST) with the options “blastn” and “--word-size 7”. The resulting blast tables were used for lowest common ancestor (LCA) assignment applying the tool blast2rma of MEGAN. The final output was visualized with the absolute read counts, using MEGAN v6.21.16.

**Figure S2: Top bacterial families in the brain tissue as inferred by different taxonomic classifiers.** DIAMOND search was performed against the NCBI non-redundant protein database (NCBI-nr), then the reads were assigned to their lowest common ancestor (LCA) using MEGAN [1]. While Kraken2 search was performed against Kraken standard database and the family-level abundances were estimated using BRACKEN [2]. MEGAN alignment tool (MALT) was ran against database of ~16,600 bacterial representative genomes [3] and MEGAN LCA was used similar to DIAMOND.


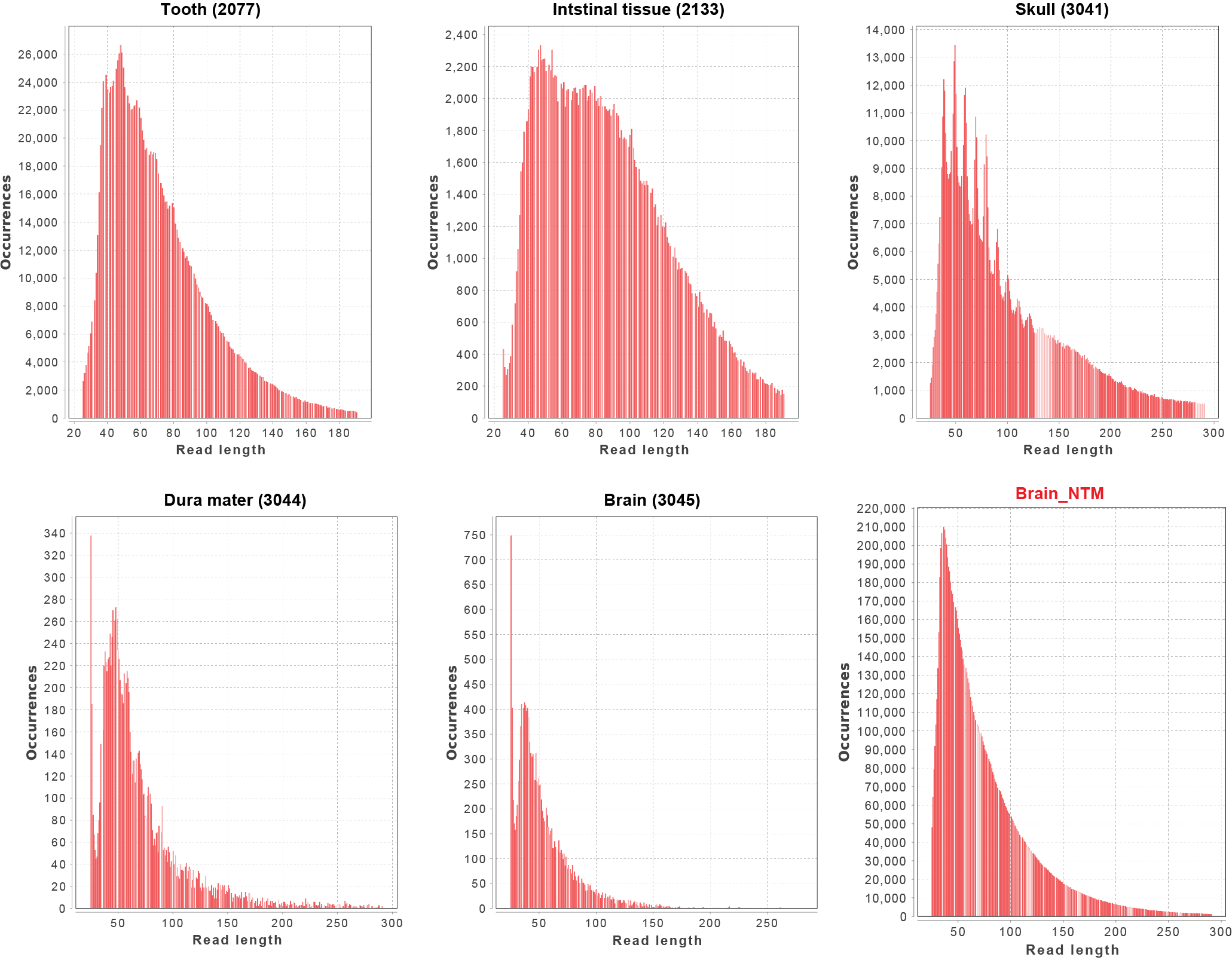


**Figure S3: Read lengths distribution of human DNA of different tissues as well as the brain NTM.** For the different body tissues (Tooth, Intestinal tissues, Skull, Dura mater, and brain), the metagenomic reads were mapped against the human reference genome (hg19), then the read lengths were calculated using MapDamage2.0 [4]. While for the brain_NTM, the brain metagenomic reads were mapped against the assembled genome of the brain_NTM, then the read lengths were calculated using DamageProfiler [5]. For further information on the samples’ origin, please refer to **Additional file 1: Table S1**.


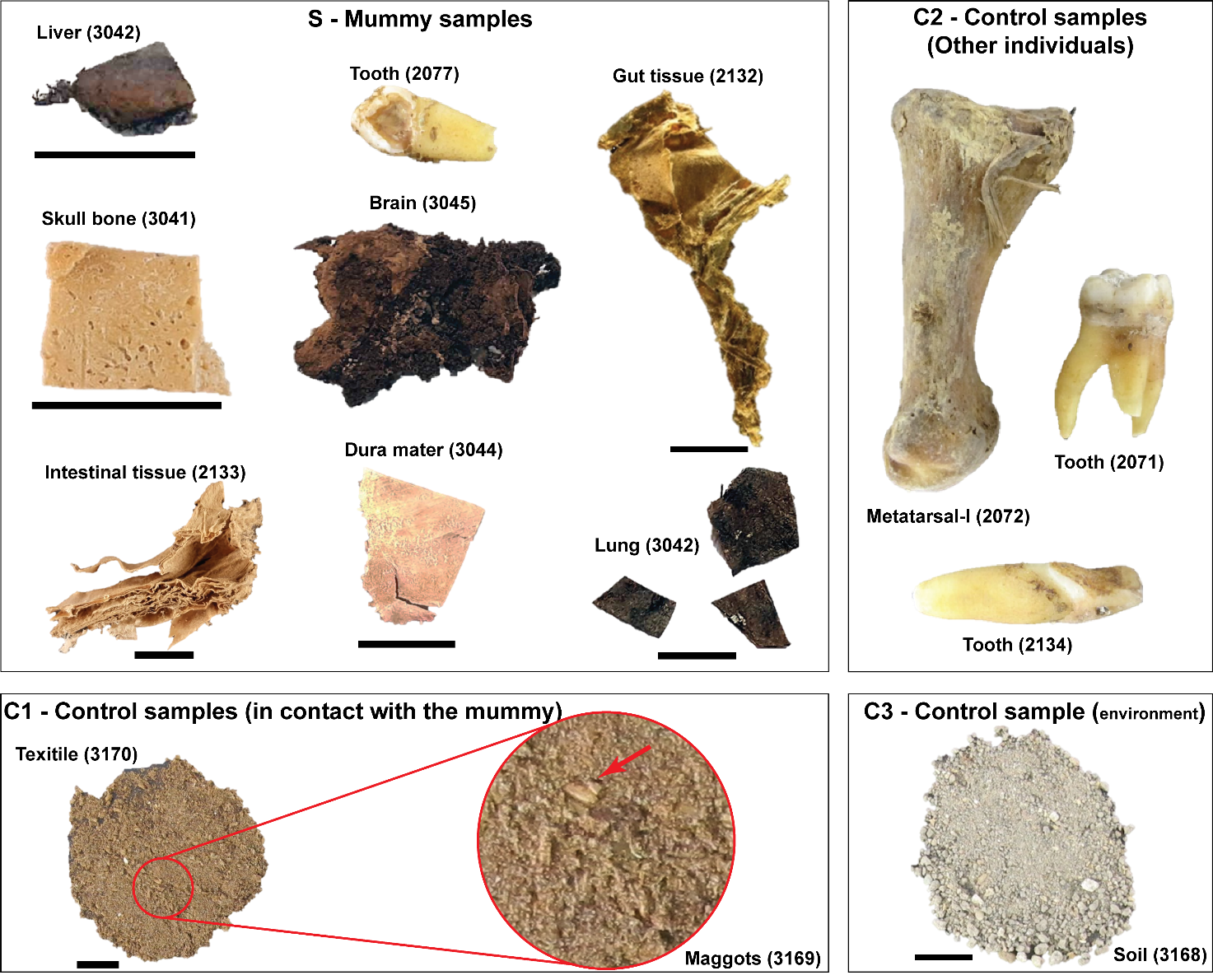


**Figure S4: Description of the analyzed samples. S**, the mummy samples are those collected from the mummy itself and assigned to particular tissues based on morphological and histological analyses [6], while the **C1** samples, are those which were collected from underneath the mummy and were in between the body and the cloths (i.e., the textile and the maggots). **C2**, the samples collected from the skeletons which were found in the other coffin in the same grave. **C3**, a soil sample collected from the soil layer that was covering the mummy, most likely was added after the reburial during the 18^th^ century. Please refer to **Additional file 1: Table S1** for further details. The black bars refer to scales of 10 mm.


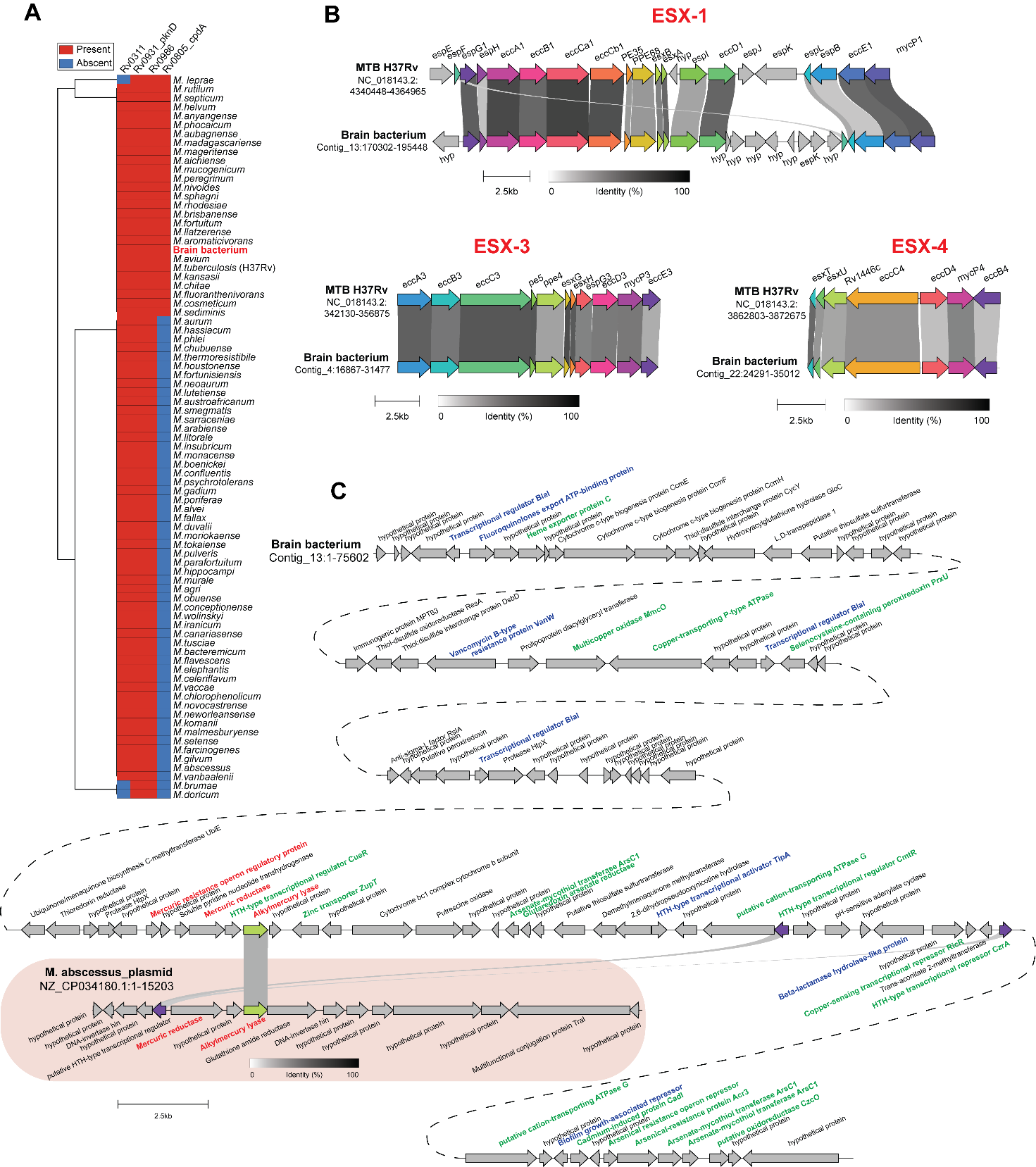
 **Figure S5: Virulence genes of the brain NTM (A)** Heatmap showing the presence/absence of genes involved in crossing Blood-Brain Barrier (BBB) and brain invasion. **(B)** Gene cluster comparison of type VII secretion systems in *Mycobacterium tuberculosis* and ACB brain bacterium. **(C)** Synteny map of the mercury resistance operon of *Mycobacterium abscessuss* compared with the mercury resistance genes (in red) of the ACB brain NTM. The map also shows the neighboring heavy metal- (in green) and antibiotics (in blue) resistance genes.


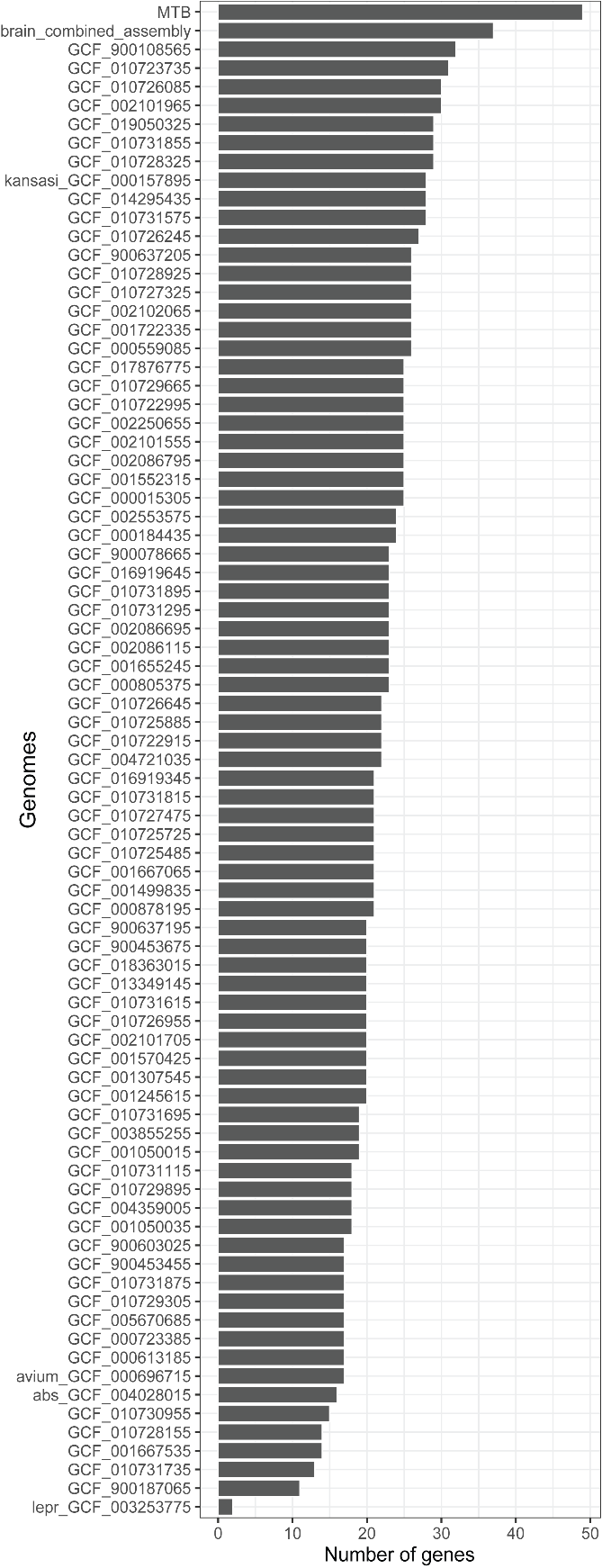


**Figure S6: Toxin/Antitoxin (TA) Systems in the analyzed mycobacterial genomes.** The horizontal bars refer to the sum of the toxin/antitoxin proteins found in the analyzed genomes.


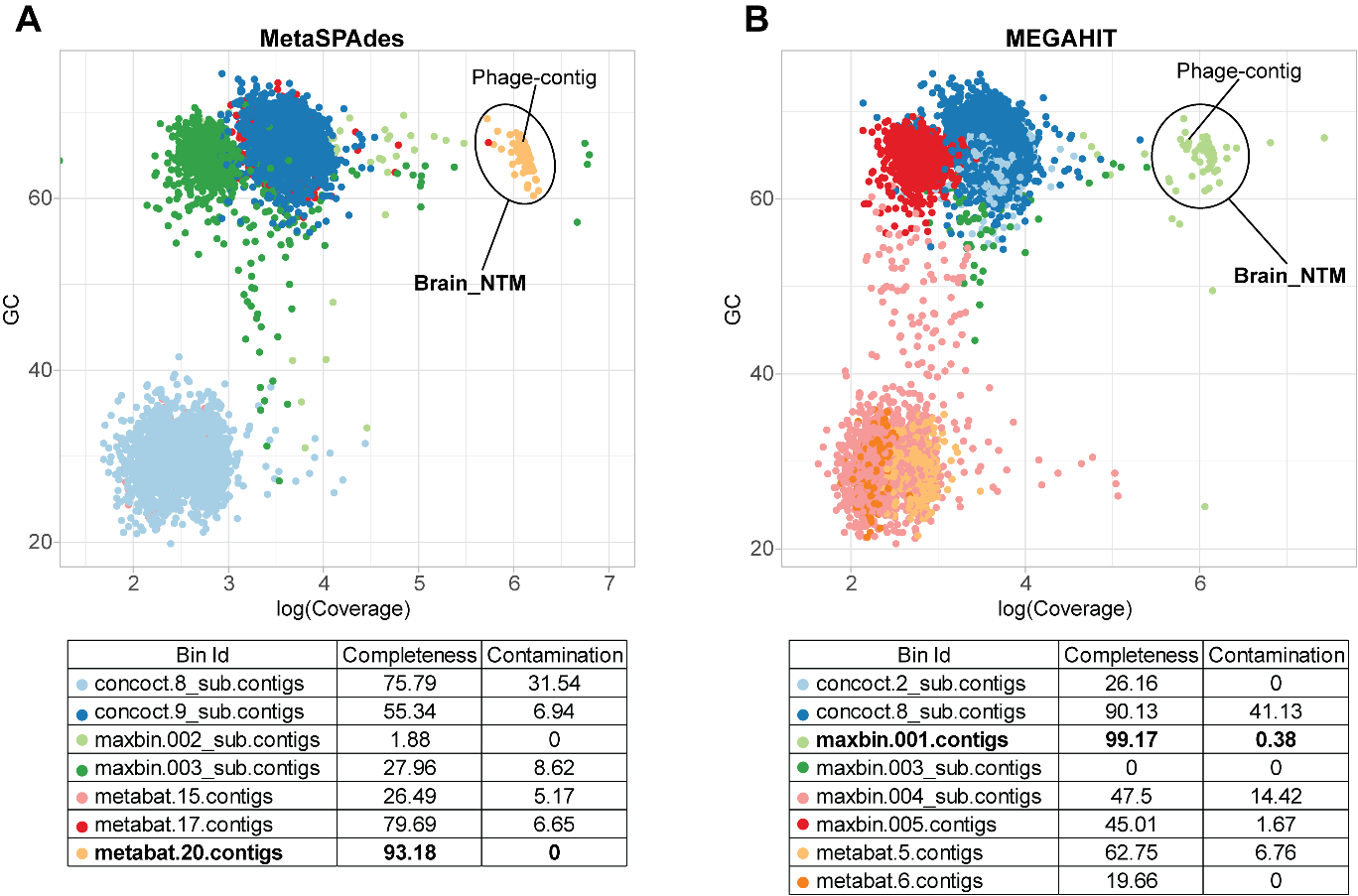


**Figure S7: Metagenomic binning of Anna Catharina Bischoff’s (ACB) brain sample.** The upper biplots show the clustering of the contigs assembled by MetaSPAdes (**A**) and MEGAHIT (**B**). The colors refer to the different bins as resulted from DAS_Tool, which combined the output of three different binners (Metabat2, MaxBin2, and CONCOCT). The circled contigs refer to the brain_NTM, and the black line inside the circle refers to the contig_38 (which contains a phage genome). The lower panel shows the completeness and the contamination of the resulting bins as estimated by CheckM. The bins in bold fonts are the brain_NTM.


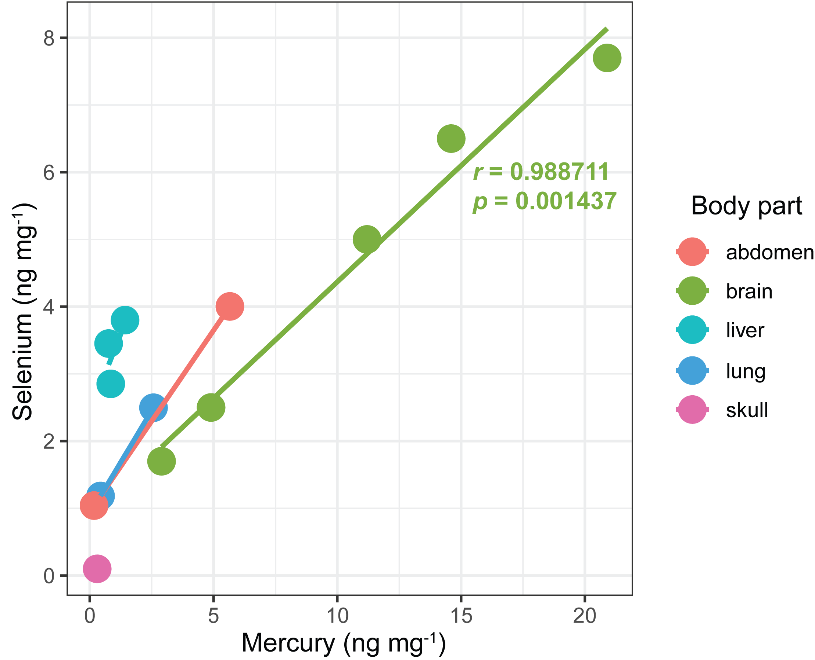


**Figure S8: Correlation analysis between concentrations of mercury (Hg) and Selenium (Se) in different body parts.**

**Supplementary references**

1. Huson DH, Beier S, Flade I, Gorska A, El-Hadidi M, Mitra S, Ruscheweyh HJ, Tappu R: **MEGAN Community Edition - Interactive Exploration and Analysis of Large-Scale Microbiome Sequencing Data**. *PLoS Comput Biol* 2016, **12**(6):e1004957.

2. Lu J, Rincon N, Wood DE, Breitwieser FP, Pockrandt C, Langmead B, Salzberg SL, Steinegger M: **Metagenome analysis using the Kraken software suite**. *Nature protocols* 2022:1-25.

3. Herbig A, Maixner F, Bos KI, Zink A, Krause J, Huson DH: **MALT: Fast alignment and analysis of metagenomic DNA sequence data applied to the Tyrolean Iceman**. *bioRxiv* 2016:050559.

4. Jónsson H, Ginolhac A, Schubert M, Johnson P, Orlando L: **mapDamage2.0: fast approximate Bayesian estimates of ancient DNA damage parameters**. *Bioinformatics* 2013, **29**(13):1682-1684.

5. Neukamm J, Peltzer A, Nieselt K: **DamageProfiler: Fast damage pattern calculation for ancient DNA**. *Bioinformatics* 2021.

6. Hotz G, Opitz-Belakhal C: **Anna Catharina Bischoff. Die Mumie aus der Barfüsserkirche**. Basel, Switzerland: Christoph Merian Verlag; 2021.
